# Levels of filaggrin degradation products are influenced by both filaggrin genotype and atopic dermatitis severity

**DOI:** 10.1111/j.1398-9995.2010.02540.x

**Published:** 2011-07

**Authors:** S Kezic, G M O’Regan, N Yau, A Sandilands, H Chen, L E Campbell, K Kroboth, R Watson, M Rowland, W H Irwin McLean, A D Irvine

**Affiliations:** 1 Coronel Institute of Occupational Health, Academic Medical CenterAmsterdam, the Netherlands; 2 The National Children’s Research Centre, Our Lady’s Children’s HospitalDublin, Ireland; 3 Department of Paediatric Dermatology, Our Lady’s Children’s HospitalDublin, Ireland; 4 Division of Molecular Medicine, University of DundeeDundee, UK; 5 Institute of Medical Biology, Agency for Science, Technology and ResearchSingapore; 6 UCD School of Medicine and Medical SciencesDublin, Ireland; 7 Department of Medicine, Trinity CollegeDublin, Ireland

**Keywords:** 2-pyrrolidone-5-carboxylic acid, FLG gene mutations, natural moisturizing factor, tyrosine, urocanic acid

## Abstract

**Background:**

Filaggrin, coded by *FLG*, is the main source of several major components of natural moisturizing factor (NMF) in the stratum corneum (SC), including pyrrolidone carboxylic acid (PCA) and urocanic acid (UCA). Loss-offunction mutations in *FLG* lead to reduced levels of filaggrin degradation products in the SC. It has recently been suggested that expression of filaggrin may additionally be influenced by the atopic inflammatory response. In this study, we investigated the levels of several breakdown products of filaggrin in the SC in healthy controls (CTRL) and patients with atopic dermatitis (AD) in relation to *FLG* null allele status. We examined the relationship between NMF (defined here as the sum of PCA and UCA) and AD severity.

**Methods:**

The SC levels of filaggrin degradation products including PCA, UCA, histidine (HIS) and tyrosine were determined in 24 CTRL and 96 patients with moderate-to-severe AD. All subjects were screened for 11 *FLG* mutations relevant for the study population.

**Results:**

The levels of PCA, UCA and HIS correlated with *FLG* genotype. Furthermore, these levels were higher in the CTRL when compared to AD patients with no *FLG* mutations. Multiple regression analysis showed that NMF levels were independently associated with *FLG* genotype and severity of disease.

**Conclusion:**

Decreased NMF is a global feature of moderate-to-severe AD; within AD, *FLG* genotype is the major determinant of NMF, with disease severity as a secondary modifier. NMF components are reliably determined by a noninvasive and relatively inexpensive tape stripping technique.

Filaggrin gene (*FLG*) loss-of-function mutations underlie ichthyosis vulgaris, a semi-dominant disorder of keratinization, and are the strongest and most widely replicated genetic risk factor for atopic dermatitis (AD) (1). We have recently shown using Raman spectroscopy that *FLG* genotype is a major determinant of natural moisturizing factor (NMF) in the stratum corneum (SC) (2, 3). Furthermore, we found that the tyrosine (TYR) levels in the palmar SC of patients with AD were positively correlated with the number of *FLG* null alleles, although the mechanism underlying elevated TYR in the carriers of *FLG* loss-of-function mutations is not yet clear.

*FLG* codes for profilaggrin, a large protein precursor comprised of 10–12 filaggrin repeats. The most abundant amino acid residues in filaggrin repeats are basic amino acids histidine (HIS) (413/4061 residues; 10.17%) and arginine (440/4061; 10.83%) and the polar residue glutamine (367/4061; 9.04%) (Fig. S1). The protein is also significantly rich in glycine (12.76%) and serine (24.06%), consistent with the structural similarity of filaggrin and related proteins such as loricrin to the variable domains of keratins, which largely consist of glycine/serine loop structures (4). Filaggrin contains fewer than average TYR residues (1.28%); however, the linker segments and carboxyl-terminal domains of profilaggrin contain dense and highly conserved TYR-rich motifs (5–7). In the later stages of epidermal differentiation, filaggrin is degraded into free amino acids and their derivatives; a major proportion of the total SC free amino acids (70–100%) are derived from filaggrin (8–10). Glutamine is further converted via a nonenzymatic process into pyrrolidone-5-carboxylic acid (PCA). PCA is highly hygroscopic and is one of the major NMF constituents (11). Histidine is deiminated to trans-urocanic acid (trans-UCA) by the catalytic action of histidase (12). Trans-UCA, which is converted to cis-UCA upon UV irradiation, functions as a major chromophore in skin and exhibits immunomodulatory effects (13). Furthermore, UCA has been suggested as a contributor to the maintenance of the acidic pH in skin, which is crucial for the activity of several key enzymes within the SC (14). Recently, we have shown that UCA and PCA exert profound effects on *Staphylococcus aureus* at physiologic concentrations (15). Filaggrin degradation products thus have multiple functions, and reduced levels as a consequence of *FLG* loss-of-function mutations may affect skin hydration, local immune responses, lipid composition (16, 17) and maintenance of epidermal homeostasis.

*In vitro* evidence has suggested that co-expression of IL-4 and IL-13, known to be upregulated in AD, can downregulate the expression of filaggrin (18). Here, we compared the levels of filaggrin degradation products in moderate-to-severe AD patients with known *FLG* mutations (*AD*_*FLG*_) and those wild type for *FLG* mutations (*AD*_*NON-FLG*_) with unaffected controls. We also investigated the relationship between severity of AD and NMF levels, defined here as the sum of PCA and UCA.

## Subjects and methods

Ninety-six unrelated Irish children with a history of moderate-to-severe AD were recruited from dedicated secondary and tertiary referral AD clinics. Twenty-five healthy children with no history of AD or atopic disease were recruited from patients attending for laser therapy of vascular birthmarks. The diagnosis and phenotyping of AD was made by experienced pediatric dermatologists. All subjects met the UK diagnostic criteria (19). Exclusion criteria from the study were patients who had received systemic therapy, such as corticosteroids or immunosuppressants in the preceeding 3 months, and patients whose ancestry was not exclusively Irish (4/4 grandparents). Detailed phenotypic data were collected and are presented in Table 1. The Nottingham Eczema Severity Score (NESS) was selected as a measure of disease severity (20). The study was conducted in accordance with Helsinki Declarations and was approved by the Research Ethics Committee of Our Lady’s Children’s Hospital, Dublin. Full written consent was obtained from all patients or their parents. Transepidermal water loss (TEWL) was measured on nonlesional skin of the extensor forearm (Tewameter 300; Courage and Khazaka Electronic GmbH, Cologne, Germany).

**Table 1:** Clinical and demographic characteristics of the cohort

*FLG* genotype	Number	Age in years *n*mean (SD)	Male gender *n*(%)	IgE *n*median (range)	NESS *n*median (range)	SCORAD* *n*, mean median (range)	TEWL *n*median (range) (g/m^2^/h)
CTRL	24	24 5.6 (4.0)	9 (38)	/	/	/	24 10.4 (6.8–10.7)
*FLG+/+*	40	34 8.1 (4.2)	19 (53)	29 599 (2–44294)	28 13 (8–15)	9 12.0 (0–36)	26 12.0 (5.2–38.3)
*FLG+/−*	37	37 8.7 (4.1)	23 (62)	35 1033 (14.9–23260)	36 12 (6–15)	19 12 (0–28)	35 14.1 (5.3–30.6)
*FLG−/−*	19	19 8.6 (4.5)	12 (63)	16 1803 (62.9–6684)	18 12 (6–15)	10 21 (0–32)	17 15.3 (8.5–30.4)
Total *AD*	96	90 8.5 (4.2)	54 (59.0)	80 1016 (2–44294)	82 12 (6–15)	38 13 (0–36)	78 13.5 (5.2–38.3)

AD, atopic dermatitis; CTRL, healthy controls; TEWL, transepidermal water loss; NESS, Nottingham Eczema Severity Score (20).

* Not available for all subjects.

### Genetic screening

All patients were screened for 11 *FLG* mutations found in the Irish population, as previously described (3). Based on screening for these 11 prevalent mutations, 58% were carriers of one or more *FLG* mutations (42%*FLG*+/+; 38%*FLG*+/−; 20%*FLG*−/−). Primers and conditions for these 11 mutations are given in detail in the Supporting information.

### Determination of filaggrin degradation products in the stratum corneum

The levels of PCA, trans- and cis-UCA, HIS and TYR were measured using a tape stripping technique from nonlesional skin of the volar forearm as previously described by Kezic et al. (21). Briefly, round adhesive tape discs (3.8 cm^2^, D-Squame; CuDerm, Dallas, TX, USA) were attached to the skin of the forearm. Each tape was applied to the volar aspect of the forearm for 10 s using a disc pressure applicator (CuDerm). The tape strip was gently removed with tweezers and stored in a closed vial at −20°C until analysis. The first strip was discarded as it may have contained dirt and remnants of cosmetic products; the second, third, fourth and fifth tape strips were applied on the same skin spot. The collected four tape strips were cut into two equal pieces. For the analysis, halves of four strips were pooled for the analysis. Before HPLC analysis, 500 μl KOH solution, 0.1 M, was added to the tape strips, followed by 2 h of continuous shaking. The alkaline extracts were neutralized with 3 μl perchloric acid, 12 M, and filtered through a 0.2-μm membrane filter. An aliquot of 50 μl was taken for the analysis of proteins by using the Bio-Rad DC protein microassay (Bio-Rad Laboratories, München, Germany) using commercially available BSA for standardization. Another 50 μl of aliquot was introduced into the HPLC system containing a 250 × 3 mm reversed-phase Prevail HPLC column (Grace/Alltech, Breda, the Netherlands). For the analysis of PCA and UCA isomers, isocratic elution was performed with 20 mM ammonium formate containing 1.5 mM tetrabutylammonium hydroxide and 1% acetonitrile at pH 7.3 (HPLC method I). The effluent was monitored at 210 nm for PCA and at 270 nm for both UCA isomers, using two sequential UV/Vis detectors, model 759A (Applied Biosystems, Carlsbad, California 92008) and model UV-975 (Jasco, Tokyo, Japan). The levels of the UCA were given as a total of the cis- and trans-isomer. Representative chromatograms obtained from the SC of patients of different *FLG* status are shown in Fig. S2. For the analysis of HIS and TYR, the HPLC analysis was modified as follows: isocratic elution was performed with 6 mM hydrochloric acid, 0.3 mM octane-1-sulfonic acid sodium salt and 1% acetonitrile at pH 3 (HPLC method II). HIS and TYR were monitored at 210 nm using a UV/VIS-detector (Jasco, model UV975). The injection volume was 4 μl. Representative chromatograms obtained from the SC of patients of different *FLG* status are shown in Fig. S3. The levels of PCA, UCA, HIS and TYR were normalized for the protein value.

### Statistical analysis

Patients were characterized, *a priori*, into three genotypes (*FLG*+/+, *FLG*+/− and *FLG*−/−), where *FLG*+/+ represents patients with no *FLG* mutations, *FLG*+/− represents patients with one *FLG* mutation, and *FLG*−/− represents patients with two *FLG* mutations. The healthy controls (CTRL) were genotyped in the same way as patients with AD; all but one control individual were wild type for *FLG* mutations and this person was excluded from the data analysis.

For testing of distribution, we used the Shapiro–Wilk’s test. In case of non-normal distribution, data were log-transformed. The means were then compared by analysis of variance (anova) followed by a *post hoc* Tukey analysis for comparison of pairwise differences between the genotype subgroups of patients with AD. For comparison of the levels of filaggin degradation products between CTRL *vs FLG*+/+, we used a Student’s one-tailed test. *P*-values <0.05 were considered statistically significant. In the figures, log-normal data are presented as geometric mean ± 95% CIs. To investigate the contribution of the number of *FLG* null mutations and severity of disease, we used multiple regression analysis with NMF (the sum of PCA and UCA) as a dependent variable and *FLG* mutations (0, 1 and 2) and disease severity as assessed by NESS and the SCORing Atopic Dermatitis (SCORAD) score as independent variables (SPSS 7.0, IBM Corporation, Somers, NY 10589).

## Results

### Filaggrin degradation components in relation to *FLG* allele status

**Figure 1:**
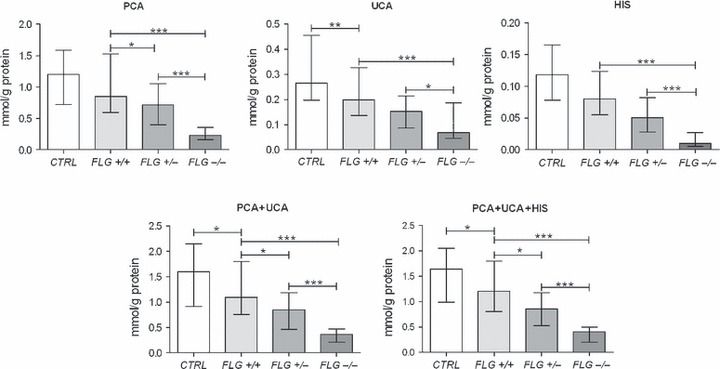
Filaggrin degradation products (geometric mean and 95% CI) in healthy controls (CTRL) and patients with atopic dermatitis (AD) in relation to *FLG* genotype. For statistic analysis, all data were log-transformed. Within AD patient subgroups, anova analysis with a *post hoc* Tukey multiple correction has been applied. To test differences between CTRL and AD_*NON-FLG*_ (*FLG*+/+), the one-tailed Student’s *t*-test was used. *P < 0.05, **P < 0.01; ***P < 0.001.

Clinical characteristics and summary data of the study cohort including *FLG* genotype are outlined in Table 1. The median values of PCA, UCA, HIS and their sum according to *FLG* genotype are shown in Table 2. Within subgroups, a range of values of filaggrin breakdown products was seen (Table 2); these values showed log-normal distribution. *Post hoc* Tukey analysis showed clearly that the levels of these filaggrin breakdown products in patients with AD were related to *FLG* status (Fig. 1).

**Table 2:** Median values (and range) of filaggrin degradation products in healthy controls (CTRL) and patients with atopic dermatitis in relation to *FLG* genotype

	PCA (mmol/g protein)	UCA (mmol/g protein)	HIS (mmol/g protein)	NMF (PCA + UCA) (mmol/g protein)	PCA + UCA + HIS (mmol/g protein)
*N*	120	120	97	120	97
CTRL	1.20 (0.43–3.83)	0.26 (0.10–1.29)	0.12 (0.02–0.23)	1.60 (0.54–4.8)	1.64 (0.74–4.64)
*FLG*+/+	0.85 (0.29–3.62)	0.20 (0.04–0.77)	0.08 (0.007–0.21)	1.09 (0.34–4.10)	1.20 (0.40–3.60)
*FLG*+/−	0.40 (0.09–2.07)	0.15 (0.03–0.45)	0.05 (0.001–0.25)	0.84 (0.19–2.34)	0.80 (0.20–1.90)
*FLG*−/−	0.23 (0.09–0.94)	0.07 (0.008–0.32)	0.01 (0.002–0.18)	0.36 (0.12–1.12)	0.40 (0.10–1.30)

HIS, histidine; NMF, natural moisturizing factor; PCA, pyrrolidone carboxylic acid; UCA, urocanic acid.

As seen from Table 1, the average age and proportion of men in patients with AD were higher than in the controls. However, in the multiple regression analysis with *FLG* status, age and sex as independent variables, sex and age did not have an effect on the NMF (*P* > 0.1).

The concentration of filaggrin degradation products is normalized by the protein amount to compensate for variable amounts of SC harvested by tape stripping. To enable comparison with literature data, we report the mean concentrations of PCA and UCA in nmol/cm^2^. The PCA levels amounted to 17.9, 14.4, 8.2 and 4.7 nmol/cm^2^ for CTRL, *FLG*+/+, *FLG*+/− and *FLG*−/−, respectively. The respective UCA levels were 4.2, 2.4, 1.8 and 1.4 nmol/cm^2^. Approximating the thickness of the SC as 10 μm, the values expressed in nmol/cm^2^ correspond to those in mM (21). The concentration of UCA found in CTRL (4.2 nmol/cm^2^, i.e^.^ 4.2 mM) is in good agreement with published results (22).

**Figure 2:**
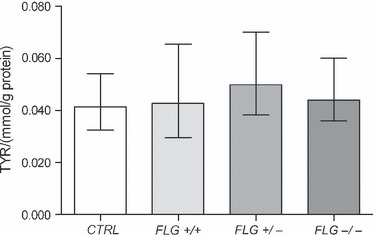
Levels of tyrosine (geometric mean and 95% CI) in relation to *FLG* genotype.

In contrast to PCA, UCA and HIS, the levels of TYR did not correlate with the *FLG* null allele status (*P* > 0.1, *post hoc* Tukey) (Fig. 2). A significant association between the levels of PCA + UCA + HIS and TYR was obtained in CTRL and *FLG* +/+ AD patients (Fig. 3A). However, the correlation between UCA + PCA + HIS and TYR was less striking in the *FLG*+/− and *FLG*−/− patients (Fig. 3B). Also, the slope of the regression line was less steep in AD_*FLG*_ patients, i.e. the level of TYR for any given concentration of PCA + UCA + HIS was higher in AD_*FLG*_ than in the CTRL or AD_*NON-FLG*_ subjects.

**3:**
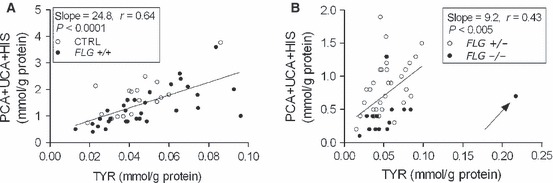
Correlation between the levels of tyrosine and pyrrolidone carboxylic acid + urocanic acid + histidine in (A) healthy controls and *FLG*+/+ and in (B) *FLG*+/− and *FLG*−/−. The outlier in (B) was excluded in the linear regression analysis. *r*, Pearson’s correlation coefficient.

### Natural moisturizing factor levels in relation to individual *FLG* mutations

Among the 11 investigated mutations, R501X and 2282del4 mutations were the most common. In the *FLG*+/− subgroup, there were 14 carriers of R501X and 15 carriers of 2282del4. Both mutations led to similar mean levels of PCA+UCA (0.87 ± 0.39 and 1.09 ± 0.62, respectively; two-sided Student’s *t*-test, *P* > 0.05). For other mutations, we had only a limited number of carriers and detailed mutation-specific expression comparisons were not possible.

### Influence of presence of atopic dermatitis and disease severity on natural moisturizing factor levels

To investigate whether the levels of filaggrin degradation products were influenced, in addition to *FLG* genotype, by the presence of disease *per se*, we compared these levels between CTRL and *FLG* wild-type patients with AD (*AD*_*NON-FLG*_; *FLG*+/+). As shown in Fig. 1 and Table 1, the levels of all NMF components tended to be higher in the CTRL subgroup when compared to the AD_*NON-FLG*_ (*FLG*+/+) subgroup: for UCA, PCA + UCA and PCA + UCA + HIS; this difference was statistically significant. To investigate the individual contribution of *FLG* null mutations and disease severity on the NMF levels, we performed multiple regression analysis in AD patients with NMF as the dependent variable and *FLG* mutations (0, 1 and 2) and disease severity as assessed either by NESS (*n* = 82) or by SCORAD (*n* = 38) as independent variables. The results showed that in addition to *FLG* mutations, severity of disease significantly contributes toward NMF levels (Table 3). There was no significant association between NMF components and TEWL or IgE in any of AD subgroups. Furthermore, the disease severity and the levels of IgE and TEWL did not statistically differ between the three AD subgroups (*P* > 0.1, anova and Tukey’s multiple comparison test). Predictably, patients with AD showed elevated TEWL when compared to CTRL (one-sided Student’s *t*-test of log-transformed data; *P* < 0.05); however, there was no difference in TEWL between AD subgroups.

**Table 3:** Natural moisturizing factor: relationship with *FLG* mutations and disease severity as assessed by NESS and SCORAD (multiple linear regressions)

	Unstandardized coefficients B (95% CI)	Standardized coefficient beta	Significance, *P*
Constant	2.248 (1.527; −2.968)		0.000
*FLG* MUT	−0.549 (−0.729; −0.369)	−0.556	0.000
NESS	−0.068 (−0.124−0.011)	−0.218	0.010
Constant	1.301 (1.02; 71.575)		0.000
*FLG* MUT	−0.333 (0.504; −0.162)	−0.512	0.000
SCORAD	−0.016 (−0.030; −0.002	−0.309	0.010

NESS, Nottingham Eczema Severity Score (19); *FLG* MUT, number of *FLG* mutations (0, 1 and 2).

## Discussion

Since the discovery of the strong association between *FLG* mutations and AD, the mechanisms through which these mutations lead to disease causation and modification have been of great interest. We have previously shown that *FLG* genotypes are the major determinants of NMF and that this association is sufficiently strong to define three SC endophenotypes within AD (3). In this study, we have replicated these findings using a SC tape stripping technique followed by HPLC as an alternative noninvasive method. We then examined the relationship between AD patients with no *FLG* mutations (AD_*NON-FLG*_) and normal controls. By very carefully defining the *FLG* status of a well-characterized collection of children with moderate-to-severe AD, we were able to further examine the relationship between AD severity, *FLG* genotype and NMF expression. We found that the two commonest *FLG* mutations had similar effects on NMF, consistent with our previous protein expression data (23) demonstrating that these mutations appear to have biologically equivalent effects. Strongly reduced levels of the filaggrin degradation products in *FLG*−/− patients (threefold decrease in *FLG*−/− patients when compared to *FLG*+/+ patients) imply that filaggrin is the major source of HIS, PCA and UCA in the SC, which is consistent with previous work (10). Thus, *FLG* genotype is the major determinant of filaggrin-derived NMF components.

Here, we demonstrated that, even in the absence of *FLG* mutations, NMF is significantly reduced in the nonlesional skin of patients with AD, implying a systemic downregulation of *FLG* expression. This is consistent with the *in vitro* findings of Howell et al. (18) and highlights the complex interplay between the skin barrier and a systemic immunologic process. Consistent with these findings, we also showed that, in AD addition to *FLG* genotype status, AD severity also has an effect on NMF levels. These findings are important and may have clinical relevance as they suggest decreased NMF is a general finding in AD and that upregulation of *FLG* expression may be beneficial to all patients with AD, rather than having an effect limited to those with *FLG* mutations. Measurement of NMF may be of value as an investigational tool to measure response to treatment before and after treatment of AD with alternative treatment strategies. HPLC of tape strip–derived material is an efficient way to measure the levels of multiple NMF components in such studies.

In contrast to our recent study reporting a markedly high positive correlation of TYR with the number of *FLG* null alleles (3), this present study observes similar levels of TYR in all investigated groups. There are several methodological differences that might have caused discrepancy in the results between these two studies. Our previous study was based on Raman spectroscopy, while here we determined TYR by using HPLC and UV spectroscopy. Both methods are highly specific, and observed differences are not likely to be caused by the use of these different detection methods. However, in the present study, TYR levels were measured in the uppermost layers of the SC from the forearm skin while in the Raman study, measurements were taken on the palmar skin and the TYR levels were averaged for the entire depth of the SC. The strong relationship between filaggrin degradation products and TYR in CTRL and *FLG*+/+ found in the present study suggests that TYR in the SC may originate from filaggrin, although filaggrin is not particularly TYR rich. In AD_*FLG*_, this relationship is less striking. Furthermore, the ratio of TYR to filaggrin degradation products is higher in AD_*FLG*_ when compared to CTRL and AD_*NON-FLG*_, suggesting either accumulation of TYR in the SC or alternative pathways in AD patients with *FLG* mutations which leads to elevated TYR levels. Further study on these TYR levels and other filaggrin degradation products in relation to skin location and SC depth is needed to clarify the elevated levels of TYR in the carriers of *FLG* loss-of-function mutations. It is quite possible that the Raman TYR signal observed in *FLG* mutation carriers, which is particularly in homozygotes (3), is because of upregulation and/or unmasking of another TYR-rich protein as part of a compensatory response to *FLG* deficiency. Proteomics analysis of SC from individuals with defined *FLG* genotypes may help resolve this issue in the future.

## Abbreviations

*FLG*: Filaggrin gene
HIS: histidine
NMF: natural moisturizing factor (defined in this study as the sum of PCA and UCA)
PCA: pyrrolidone carboxylic acid
SC: stratum corneum
TEWL: transepidermal water loss
TYR: tyrosine
UCA: urocanic acid

## Supporting Information

Data S1 FLG genotyping.

**Figure S1.** Amino acid composition of human profilaggrin. **Figure S2.** The representative HPLCchromatograms(HPLC method I)of the extracts of the SC of patientsof different *FLG* status and of the standard solution ofPCA,trans- and cis-UCA (25,25 and 75 μM, respectively). **Figure S3.** The representative HPLCchromatograms(HPLC method II)of the extracts of the SC of patientsof different *FLG* status and of the standard solution of HISand TYR (20 and 25 μM, respectively).
