# Supplementary material for: Levels of filaggrin degradation products are influenced by both filaggrin genotype and atopic dermatitis severity
Source: Allergy. 2011 Jul;66(7):934–40. doi: 10.1111/j.1398-9995.2010.02540.x (PMC3586662; doi:10.1111/j.1398-9995.2010.02540.x)
Supplement: Data S1 — FLG genotyping. [file all0066-0934-sd1.doc]

**FLG genotyping**

FLG mutations R501X, 2282del4, R2447X and S3247X were screened by Taqman allelic discrimination assays modified from Palmer *et al*, Nat Gen. 38, 441-446, (2006) and Sandilands *et al*, Nat Gen. 39, 650-654 (2007). All assays were performed using the same conditions (1 cycle at 50°C for 2 minutes followed by 1 cycle at 95°C for 10 minutes then 40 cycles of 95°C 15 sec; 60°C 1 minute) and were run on an Applied Biosystems 7900HT Fast Real-Time PCR system. R501X was screened using forward primer 5’ CAC TGG AGG AAG ACA AGG ATC G 3’ and reverse primer 5’ CCC TCT TGG GAC GCT GAA 3’ and probes for wildtype (VIC-CAC GAG ACA GCT C) and mutant (6-FAM-CAT GAG ACA GCT CC) alleles. 2282del4 was screened using forward primer 5’ CCA CTG ACA GTG AGG GAC ATT CA 3’ and reverse primer 5’ GGT GGC TCT GCT GAT GGT GA 3’ and probes for wildtype (6-FAM- CAC AGT CAG TGT CAG GCC ATG GAC A)

and mutant (VIC-AGA CAC ACA GTG TCA GGC CAT GGA CA) alleles. R2447X was screened using forward primer 5’ CAC GTG GCC GGT CAG CA 3’ and reverse primer 5’ TCC TGA CCC TCT TGG GAC GT 3’ and probes for wildtype (VIC-CAC GAG ACA GCT C) and mutant alleles (6-FAM-CAT GAG ACA GCT CC). S3247X was screened using forward primer 5’ CCA GAA ACC ATC GTG GAT CTG 3’ and reverse primer 5’ TGC CTG ATT GTC TGG AGC G 3’ and probes for wildtype (6-FAM-CAG TCA AGG CAC GG) and mutant (VIC-AGC AGT AAA GGC ACG) alleles .

FLG mutations S1040X, G1139X and R3419X were screened by restriction enzyme digest of PCR products. S1040X was screened by restriction digest of a 375bp PCR product (forward primer 5’ CCAGACAATCAGGAACTCC 3’, reverse primer 5’ ATGAGTGCTCACCTGGTAGAT 3’). PCR conditions were 94°C for 3 minutes followed by 34 cycles of 94°C 30sec; 62°C 30sec; 72°C 1 minute and a final step of 72°C for 5 minutes. Mutation S1040X creates a *BtsI* site, digestion with this enzyme yields fragments of **251bp** and **124bp** (mutant allele) whereas the wildtype allele is uncut (375bp). G1139X was screened by restriction digest of a 653bp PCR product (forward primer 5’ GATAGTGAGGGACATTCAGAGGAG 3’, reverse primer 5’ CAGACAACCTCTCGGAGTCGTCT 3’). PCR conditions were 94°C 3 minutes followed by 34 cycles of 94°C 30sec; 62°C 30sec; 72°C 1 minute and a final step of 72°C for 5 minutes. Mutation G1139X abolishes a *TspRI* site, digestion with this enzyme yields fragments of 393bp, 157bp, 51bp, 31bp, 15bp and 6bp (wildtype allele) whereas the mutant allele generates fragments of **550bp**, 51bp, 31bp, 15bp and 6bp. R3419X was screened by restriction digest of a 332bp PCR product (forward primer 5’GCCCATGGGCGGACCAGGA 3’, reverse primer 5’GCTTCATGGTGATGCGACCA 3’). PCR conditions were 94°C 3 minutes followed by 34 cycles of 94°C 30sec; 61°C 30sec; 72°C 1 minute and a final step of 72°C for 5 minutes. FLG mutation R3419X creates an *NlaIII* site and digestion with this enzyme yields fragments of 307bp, 14bp, 7bp and 4bp (wildtype allele) whereas the mutant allele produces fragments of **252bp**, **55bp**, 14bp, 7bp and 4bp.

FLG mutations Y2092X was screened by direct sequencing of a 574bp PCR product (forward primer 5’ CA CAG TCA GTG TCA GCA CAG 3’, reverse primer 5’ GGC TAA CAC TGG ATC CCC GGG 3’). PCR conditions were 94°C for 3 minutes followed by 34 cycles of 94°C 30sec; 62°C 30sec; 72°C 1 minute and a final step of 72°C for 5 minutes.

Mutation 3702delG was screened by sizing of fluorescently-labelled PCR products using methods described previously (Sandilands et al, J. Invest. Derm. 126(8):1770-1775, 2006).
