# Supplementary material for: Levels of filaggrin degradation products are influenced by both filaggrin genotype and atopic dermatitis severity
Source: Allergy. 2011 Jul;66(7):934–40. doi: 10.1111/j.1398-9995.2010.02540.x (PMC3586662; doi:10.1111/j.1398-9995.2010.02540.x)
Supplement: Supplementary file 2 — Figure S1. Amino acid composition of human profilaggrin. Figure S2. The representative HPLCchromatograms(HPLC method I)of the extracts of the SC of patientsof different FLG status and of the standard solution ofPCA,trans- and cis-UCA (25,25 and 75 μM, respectively). Figure S3. The representative HPLCchromatograms(HPLC method II)of the extracts of the SC of patientsof different FLG status and of the standard solution of HISand TYR (20 and 25 μM, respectively). [file all0066-0934-sd2.doc]

**Figure 1S**

**Amino acid composition of human profilaggrin.**

The amino acid composition of the 4061 amino acid human profilaggrin polypeptide (SwissProt accession number P20930, derived from the human RefSeq genome sequence for *FLG*, GenBank accession number NM_002016) was plotted against the average amino acid composition across the human proteome (genome.ucsc.edu). Grey bars = average amino acid composition of human proteome; Red = most significantly over- or under-represented amino acids; Blue or blank = not significantly different from average.

**Reference**

Korge BP, Gan S-Q, McBride OW, Mischke D, Steinert PM (1992) Extensive size polymorphism of the human keratin 10 chain resides in the C-terminal V2 subdomain due to variable numbers and sizes of glycine loops. Proc Natl Acad Sci USA 89:910-4.


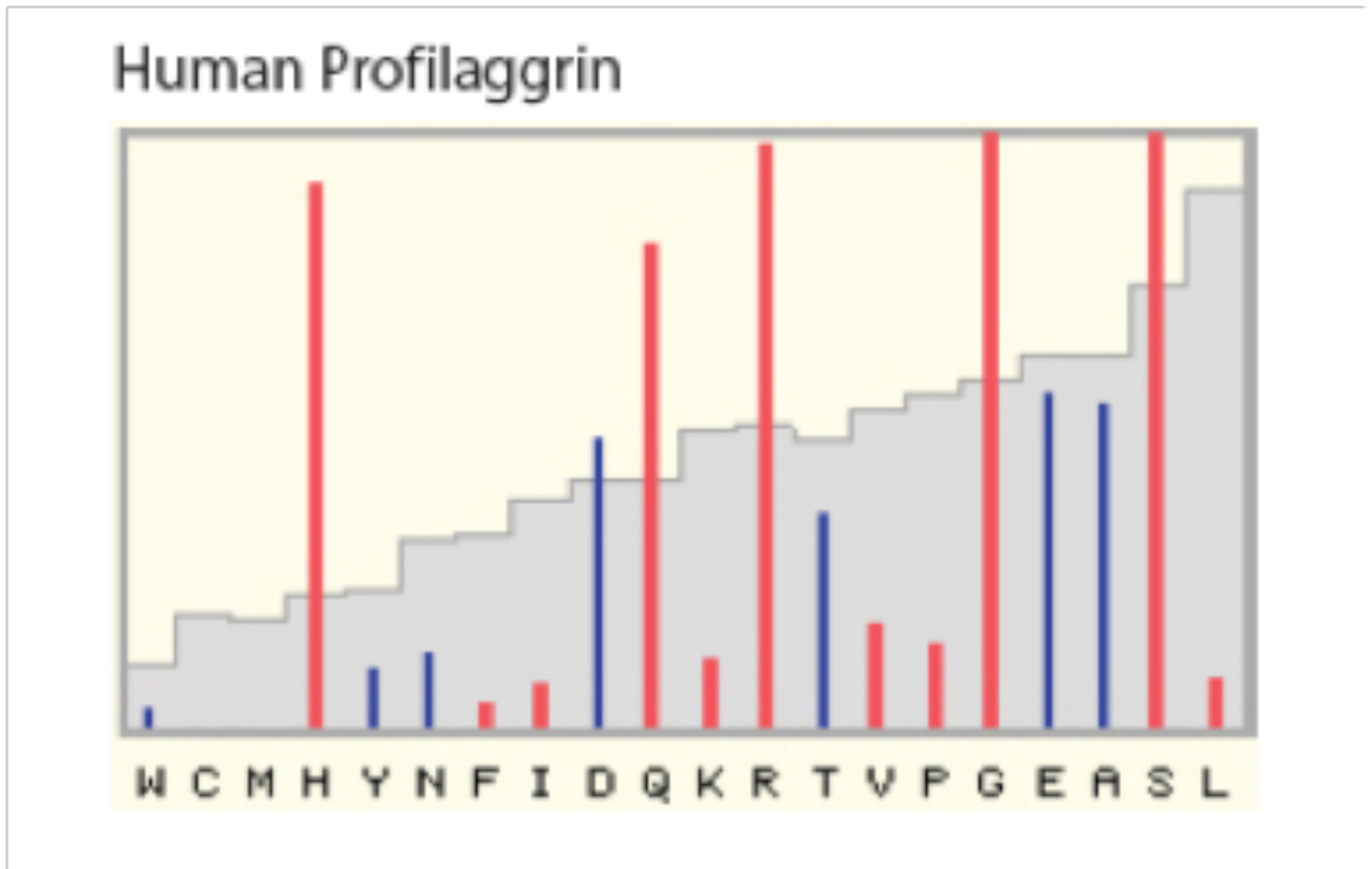


Fig 2S


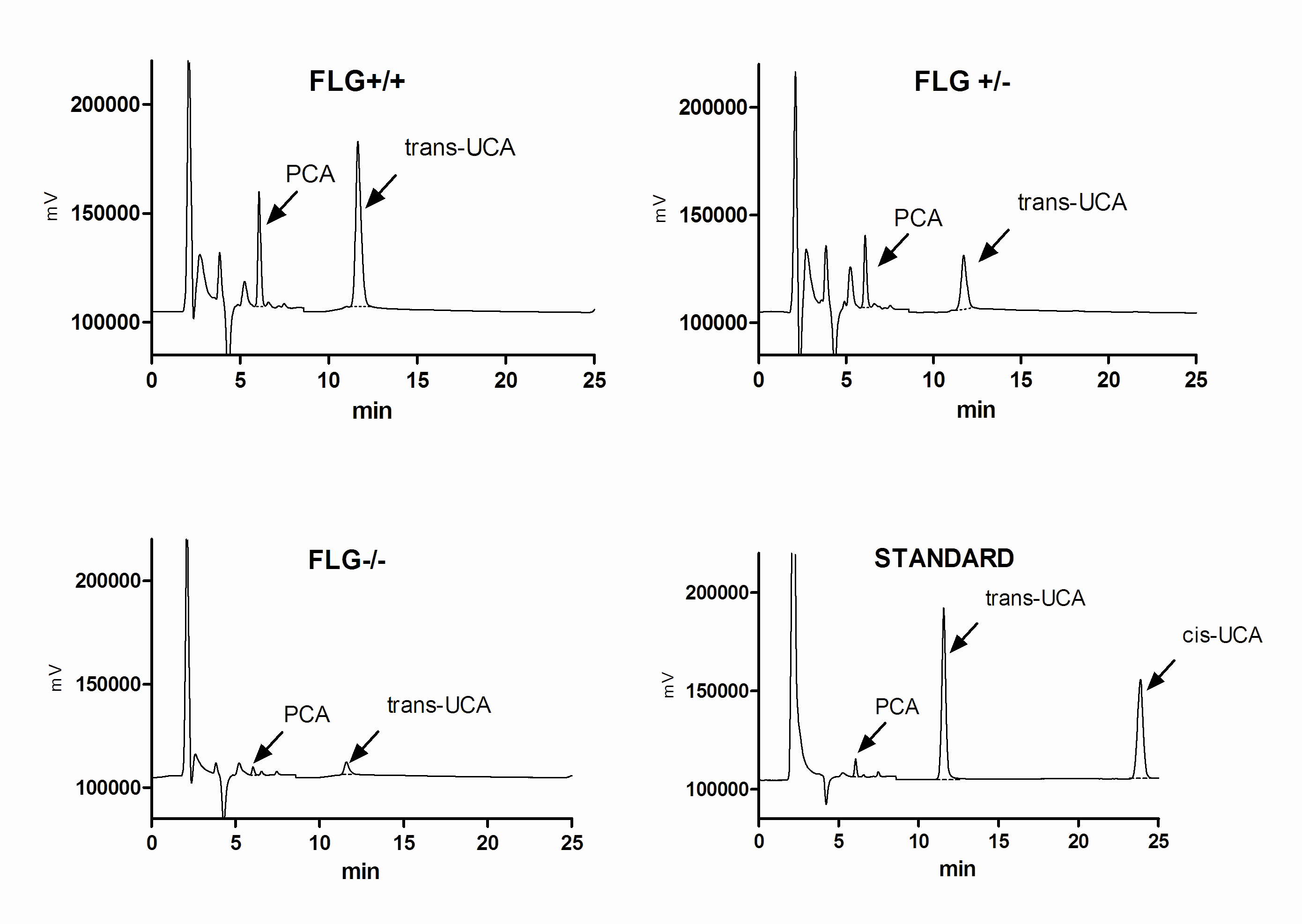


Fig. 2S. The representative HPLC chromatograms (HPLC method I) of the extracts of the SC of patients of different *FLG* status and of the standard solution of PCA, trans- and cis-UCA (25, 25 and 75 µM, respectively). *FLG* +/+ designates a homozygote wildtype, *FLG*+/- a heterozygote null allele *and FLG*-/- a patient homozygous for null alleles.

Fig 3S.


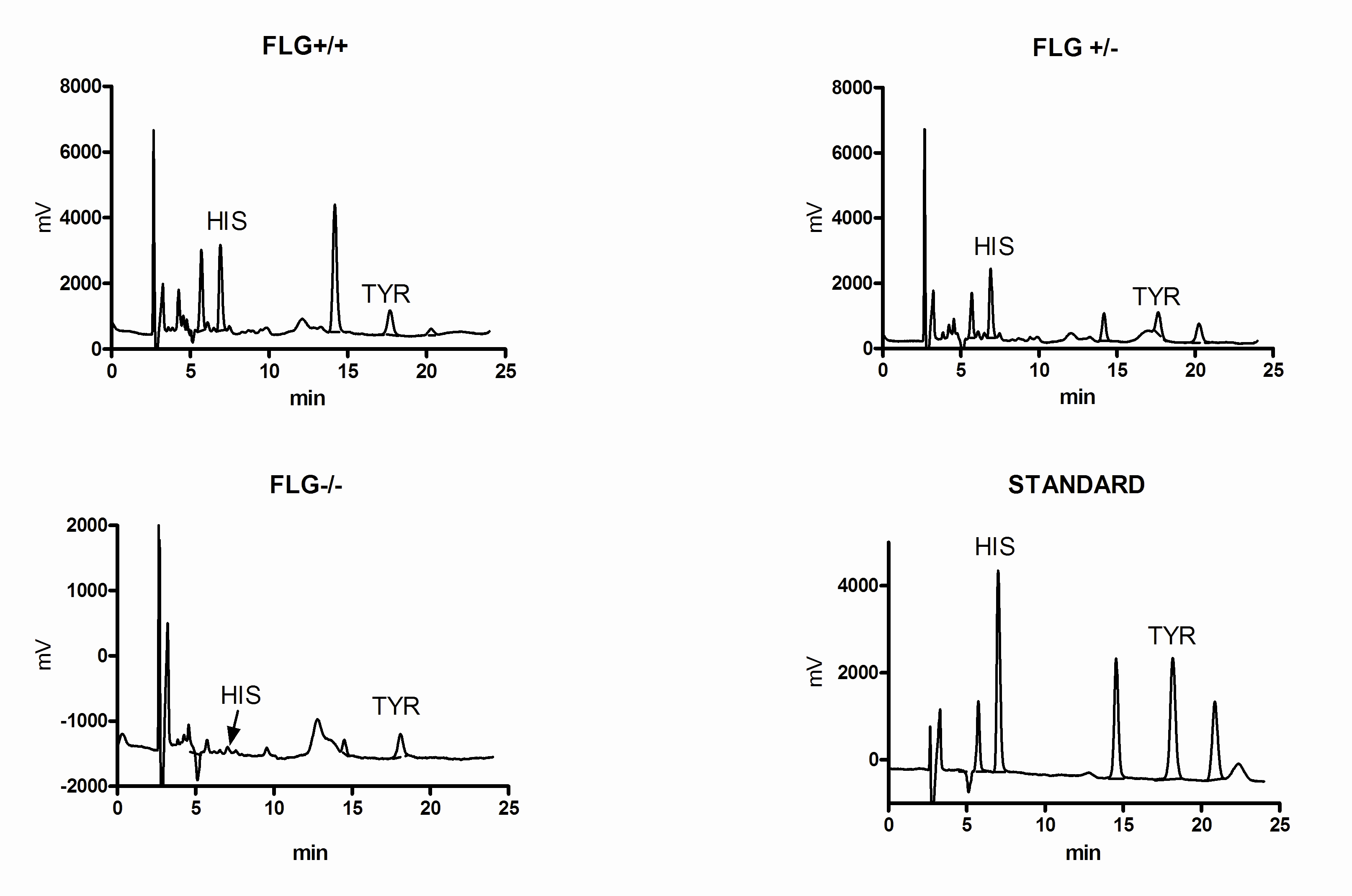


Fig. 3S. The representative HPLC chromatograms (HPLC method II) of the extracts of the SC of patients of different *FLG* status and of the standard solution of HIS and TYR (20 and 25 µM, respectively). *FLG* +/+ designates a homozygote wildtype, *FLG*+/- a heterozygote null allele *and FLG*-/- a patient homozygous for null alleles.
